# Knowledge domains and hotspots of the association between hypertension and noise: a bibliometric analysis and visualization study from 2003 to 2023

**DOI:** 10.3389/fcvm.2025.1492051

**Published:** 2025-03-25

**Authors:** Tianqi Wu, Siming Zhang, Zhou Zhang, Jun Pu, Ruizi Liu, Tianyi Yuan, Xu Chen, Songnian He, Qingyu Hao, Jue Gu

**Affiliations:** ^1^ Department of Cardiology, Affiliated Hospital of Nantong University, Medical School of Nantong University, Nantong University, Nantong, China; ^2^ Cancer Research Center Nantong, Nantong Tumor Hospital & Affiliated Tumor Hospital of Nantong University, Nantong, China; ^3^ Institute of Molecular Biomembrane and Glycobiology, Tohoku Medical and Pharmaceutical University, Sendai, Japan; ^4^ Department of Clinical Laboratory, Central Laboratory, Xishan People's Hospital of Wuxi City, Wuxi, China; ^5^ Department of Cardiology, Infectious Disease Hospital of Heilongjiang Province, Harbin, China

**Keywords:** noise, hypertension, bibliometrics, VOSviewer, CiteSpace

## Abstract

**Objectives:**

Noise pollution has become an increasingly severe environmental issue in modern society and has been recognized for its adverse effects on cardiovascular diseases. Hypertension induced by noise exposure has garnered significant research interest and a large quantity of studies have been conducted. This study aims to employ bibliometric methods to comprehensively review the literature on the relationship between noise exposure and hypertension and to analyze the current state of research by identifying key areas of focus while also exploring future trends in this field.

**Methods:**

The bibliometric analysis was conducted using the Web of Science Core Collection (WoSCC) database. The search query included terms related to noise pollution and hypertension. The timeframe for the search was from 2003 to 2023. Data analysis and visualization were performed using VOSviewer, CiteSpace, Scimago Graphica, and Rtools, focusing on publication trends, citation metrics, explosive intensity, and collaborative networks. Pajek was used to adjust pictures.

**Results:**

The bibliometric analysis showed a notable rise in research output on the relationship between noise pollution and hypertension. The United States led in the number of publications, with China and Germany coming next. The study identified several key contributors, with Muenzel Thomas being the most prolific author, followed by Daiber Andreas and Pershagen Goran. Institutionally, Johannes Gutenberg University Mainz emerged as the leading institution in terms of publications, followed by Karolinska Institute. Collaborative networks among institutions highlighted significant international cooperation, with extensive collaborations observed, particularly between European and North American institutions. The study also pinpointed research hotspots and emerging trends through keyword analysis. Key areas of focus included the mechanisms linking noise exposure to hypertension, the impact of noise on cardiovascular health, and the role of environmental stressors.

**Conclusions:**

This study advances our understanding of noise-induced hypertension's physiological and biological mechanisms, emphasizing the need for continued research. The research underscores the necessity of addressing noise pollution as a significant public health concern.

## 1 Introduction

Noise, a pervasive environmental stressor, is increasingly recognized not only for its disruption of daily human activities but also for its significant impact on public health. The detrimental health impacts of environmental noise pollution extend far beyond auditory damage, positioning it as a modifiable risk factor for cardiovascular morbidity and mortality (1, 2). Among its many adverse effects, its contribution to hypertension has become a growing concern in recent years (3–5). Noise pollution, originating from sources such as road traffic, railways, and aviation, has been shown to induce chronic stress and trigger physiological responses that elevate blood pressure (6, 7). Hypertension is the leading cause of cardiovascular disease and premature death worldwide, with its prevalence increasing in recent years (8). It is estimated that around 1.1 billion people globally suffer from hypertension, and the number is still increasing (9).Given the growing body of evidence linking noise pollution to hypertension, it is crucial to understand the broader scope of research surrounding this issue.

To gain deeper insights into the current state of knowledge and identify key trends, a bibliometric analysis is an effective tool. Bibliometric analysis is a quantitative method used to evaluate the impact and trends of scientific research in a specific field. This technique utilizes various statistical and mathematical tools to analyze research articles' publication patterns, citation frequencies, and authorship data. By examining metrics such as the number of publications and citation counts, bibliometric analysis provides insights into the productivity, influence, and collaborative networks of researchers and institutions (10–12). Herein, a bibliometric analysis was conducted to investigate the relationship between noise and hypertension, based on extensive studies from 2003 to 2023. This comprehensive approach allows researchers to pinpoint key areas of interest, track the development of new trends, and recognize the most impactful studies. Doing so provides valuable insights that can help shape future research agendas and inform policy decisions aimed at addressing the health impacts of noise on hypertension, giving useful guidance for future endeavors.

## 2 Materials and methods

### 2.1 Data collection

**Figure 1:**
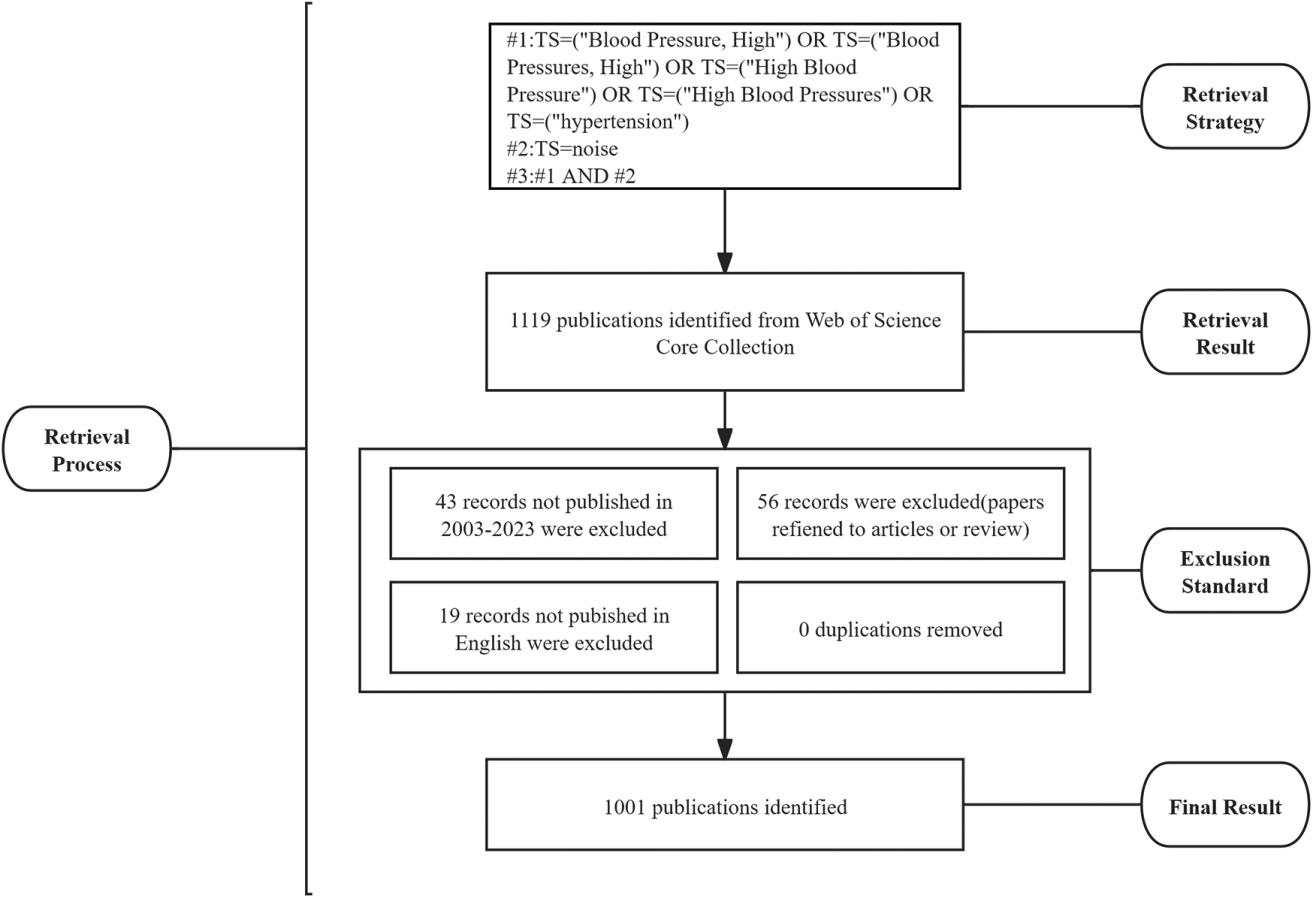
Data retrieval process Flowchart. #, connection character; TS, topic search.

On 18 July 2024, we retrieved a total of 1,119 records from the Web of Science Core Collection (WoSCC) dataset using the search terms Topic: (TS = (“Blood Pressure, High”) OR TS = (“Blood Pressures, High”) OR TS = (“High Blood Pressure”) OR TS = (“High Blood Pressures”) OR TS = (“hypertension”)) AND [TS = (noise)]. To guarantee the study's credibility and accuracy, three researchers worked together to retrieve and rigorously screen the data. We excluded 43 records not published from 2003 to 2023, 56 meeting abstracts, editorial materials, letters, corrections, and reprints, as well as 19 papers not published in English. In total, 1,001 articles or review articles were included in this study. The full records and cited references of these 1,001 articles were exported in plain text format. Figure 1 illustrates the process of data collection.

### 2.2 Data analysis and visualization

We used VOSviewer version 1.6.20 to extract information on authors, research institutions, countries, citations, and keywords from plain text files downloaded from the Web of Science (WOS) database. This data was then used to create a visual network diagram. The primary bibliometric techniques employed in this study included co-authorship analysis, co-occurrence analysis, and co-citation analysis to determine the topics and research hotspots in the literature. CiteSpace software 6.3.R1 was utilized to visualize and analyze trends and patterns in the scientific literature. This software facilitated the identification of critical paths and pivotal points within the research network, providing further insights into the temporal development of research topics. R software (version 4.3.2, Auckland University, New Zealand, https://www.r-project.org/) was employed for trend diagram drawing and keywords visualization analysis. This software played a crucial role in handling large datasets and generating insightful visualizations, enabling a comprehensive analysis of the data. Scimago Graphica (version 1.0.43) was used to draw the map of the co-authorship network of countries/regions. Pajek was used to adjust pictures.

## 3 Results

### 3.1 Publication trends

**Figure 2:**
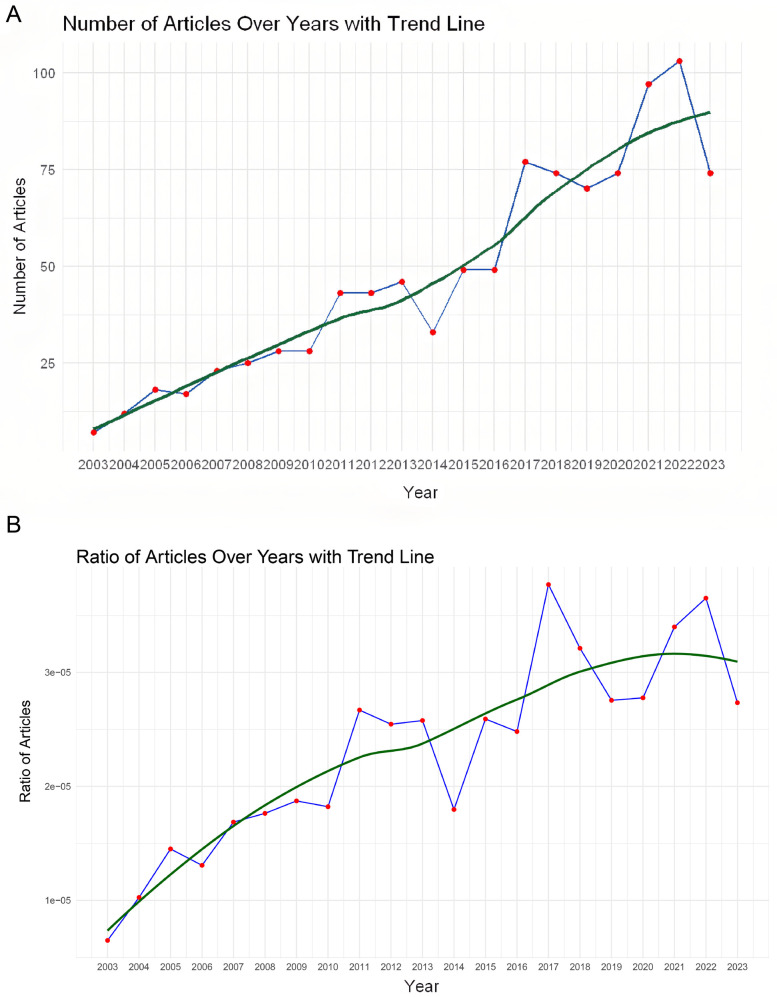
Number of publications and citations from 2003 to 2023. **(A)** The total number of publications each year, with a trend line fitted using lasso regression to capture the overall publication growth trend. **(B)** The total number of citations each year, illustrating the citation trend.

Annual publication volume serves as a significant indicator revealing the developing trend or pace of a certain field. Herein, we drew a valid graph using R software to give a valid illustration of the publication volume. Figure 2A shows the worldwide number of publications over the years and a trend line using lasso regression. Figure 2B shows the ratio of articles over the years. This ratio was calculated by dividing the number of publications on noise and hypertension by the total number of publications in the entire research field for each year. The overall trend demonstrates a steady annual increase in the volume of relevant literature from 2003 to 2023.In recent years, the annual number of relevant publications has consistently surpassed 75, while the ratio of articles has steadily increased, as reflected by the trend line. This increase in publications can reflect a significant expansion of knowledge and advancements within the field, possibly due to breakthroughs and technological advancements or greater collaboration among researchers, institutions, or countries, leading to more outputs, indicating rising awareness and concern about the health impacts of environmental noise.

### 3.2 Distribution of countries and regions

**Figure 3:**
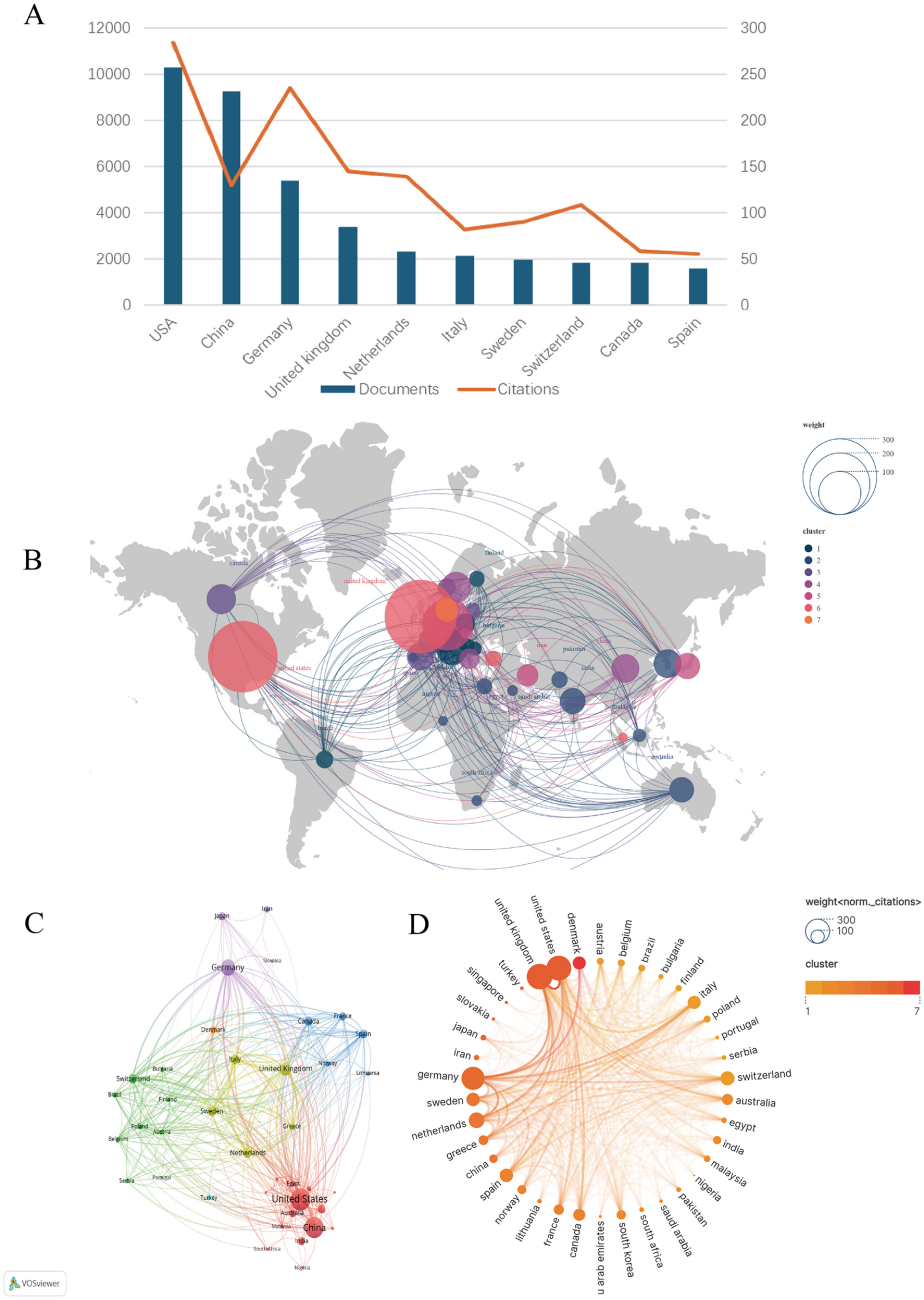
Distribution of countries and regions. **(A)** The top 10 countries ranked by the total number of publications and their corresponding citation counts, highlighting the most influential contributors in the field. **(B)** Cluster analysis of contributing countries, generated using Scimago Graphica. **(C)** Network visualization of countries and regions, each node represents a country, with node size proportional to the number of publications produced by that country. The connections between nodes indicate collaborative relationships between countries. **(D)** Map of international collaborations, illustrating co-authorship relationships between countries.

A total of 71 countries contributed to the research on the relationship between noise and hypertension. Figure 3A; Table 1 display the top 10 countries related to this topic based on the volume of publications. As illustrated in the graph, a total of 257 publications were from the USA with 11,350 citations, making the USA the leading country in the field of noise and hypertension. Although China had the second most publications, it only had the fifth most citations, indicating that it needed further improvement in article quality. Germany ranked third in publications but ranked second in citations. The economic development of a country often correlates with its ability to contribute to global research. Developed countries like the USA and Germany have the infrastructure and funding needed to support high-quality research. Conversely, while China is rapidly advancing in research output, further investment in enhancing the quality of research in specific fields such as noise and hypertension may be necessary to boost its citation impact. Figures 3C,D further showed the contribution and cooperation among countries. Figure 3D takes into account the potential bias introduced by country size. After controlling for the influence of publication scale, the United States continues to show the broadest and most prominent collaborative networks in this research field, maintaining a central role in the global scientific efforts. The result also reveals that the USA, UK, and Germany exhibit significantly higher normalized citations, reinforcing their leading positions in the field, highlighting the depth of their scientific and research cooperation.

**Table 1:** Top 10 countries/regions with the highest number of published articles.

Rank	Country	Documents	Citations	Year (start of research)	Centrality
1	United States	257	11,350	2003	0.38
2	China	231	5,186	2005	0.21
3	Germany	135	9,393	2003	0.17
4	United Kingdom	85	5,777	2003	0.2
5	Netherlands	58	5,555	2005	0.06
6	Italy	53	3,265	2005	0.16
7	Sweden	49	3,614	2005	0.13
8	Switzerland	46	4,343	2005	0.07
9	Canada	46	2,339	2003	0.04
10	Spain	40	2,200	2010	0.08

### 3.3 Author and co-author analysis

**Figure 4:**
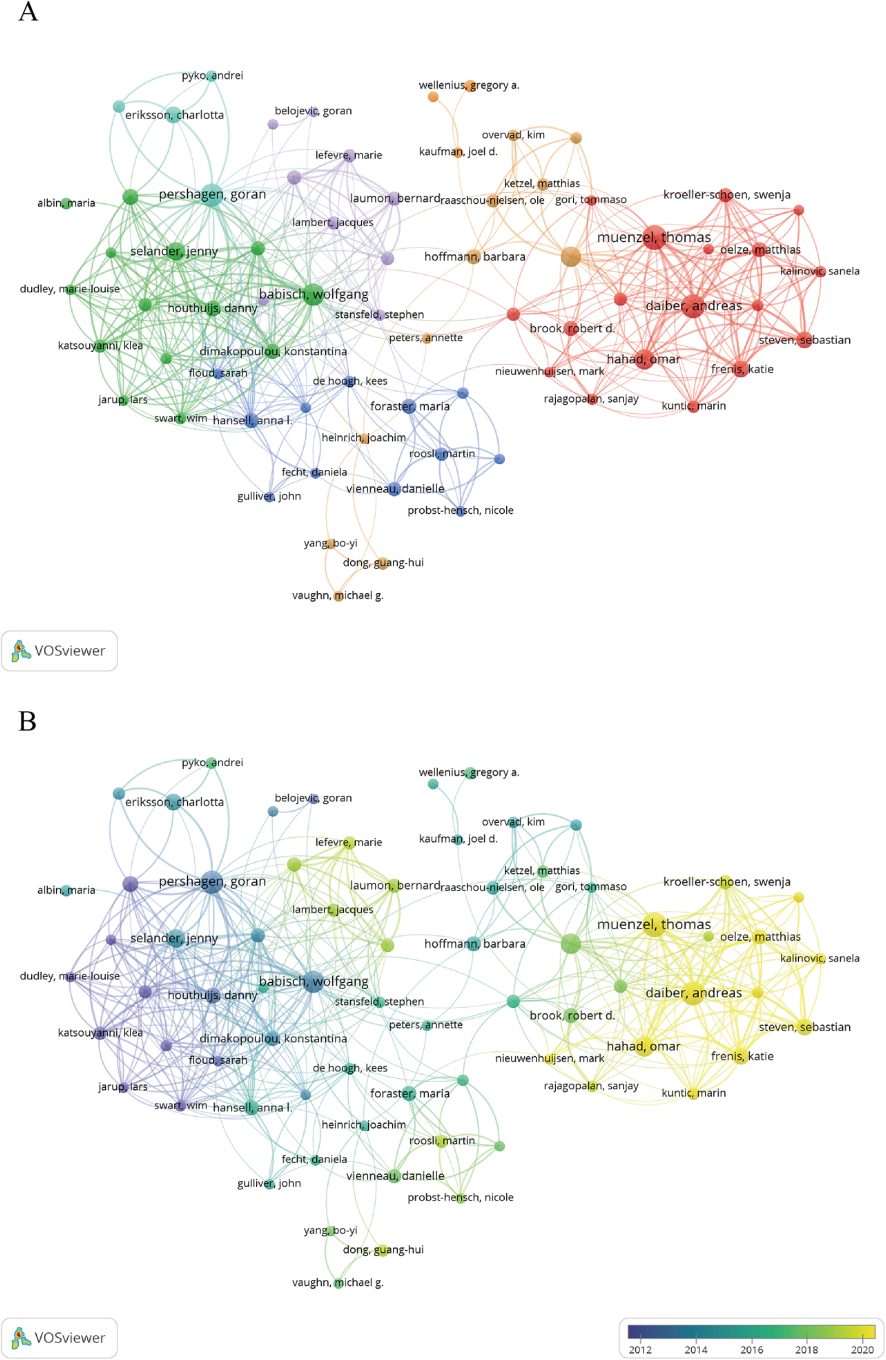
Author co-citation visualization map. Circle size reflects the author's total citations. Line thickness indicates the strength of co-citation relationships, with thicker lines denoting stronger associations. **(A)** Clustering of authors based on co-citation patterns, with colors representing different author groups. **(B)** Temporal changes in co-citation relationships, with colors indicating the evolution of these relationships over time.

Analyzing the most prolific or cited authors in a certain field helps scholars and researchers identify leading experts and significant contributors. This allows them to follow the latest research and understand hot topics and future trends in the field. According to our analysis results, more than 4,500 researchers participated in research about hypertension and noise. Among them, Muenzel Thomas (*n* = 27) ranked first in publications with 27 documents and 2,001 citations. Followed by Daiber Andreas (*n* = 26) and Pershagen Goran (*n* = 24). Babisch Wolfgang (*n* = 22) has the most citations of 3,694 (Table 2). We then utilized VOSviewer to visualize author clusters, focusing on those with over five publications. Authors with related research topics were grouped into clusters, each marked by a distinct color (Figure 4). Within the red cluster, Pershagen Goran and Babisch Wolfgang have extensive collaborations with other authors and are among the most influential contributors. Muenzel Thomas and Steven Sebastian are recognized as influential authors in the green cluster.

**Table 2:** Top 10 authors with the highest number of published articles.

Author	Documents	Citations
Muenzel Thomas	27	2,001
Daiber Andreas	26	1,572
Pershagen Goran	24	2,254
Babisch Wolfgang	22	3,694
Hahad Omar	18	673
Houthuijs Danny	11	1,139
Cadum Ennio	9	847
Dimakopoulou Konstantina	10	642
Steven Sebastian	13	922
Frenis Katie	12	398

In recent years, authors like Muenzel Thomas, Daiber Andreas, Hahad Omar have showed up with high publication volume, leading the developing pace of this area.

### 3.4 Institutions

**Figure 5:**
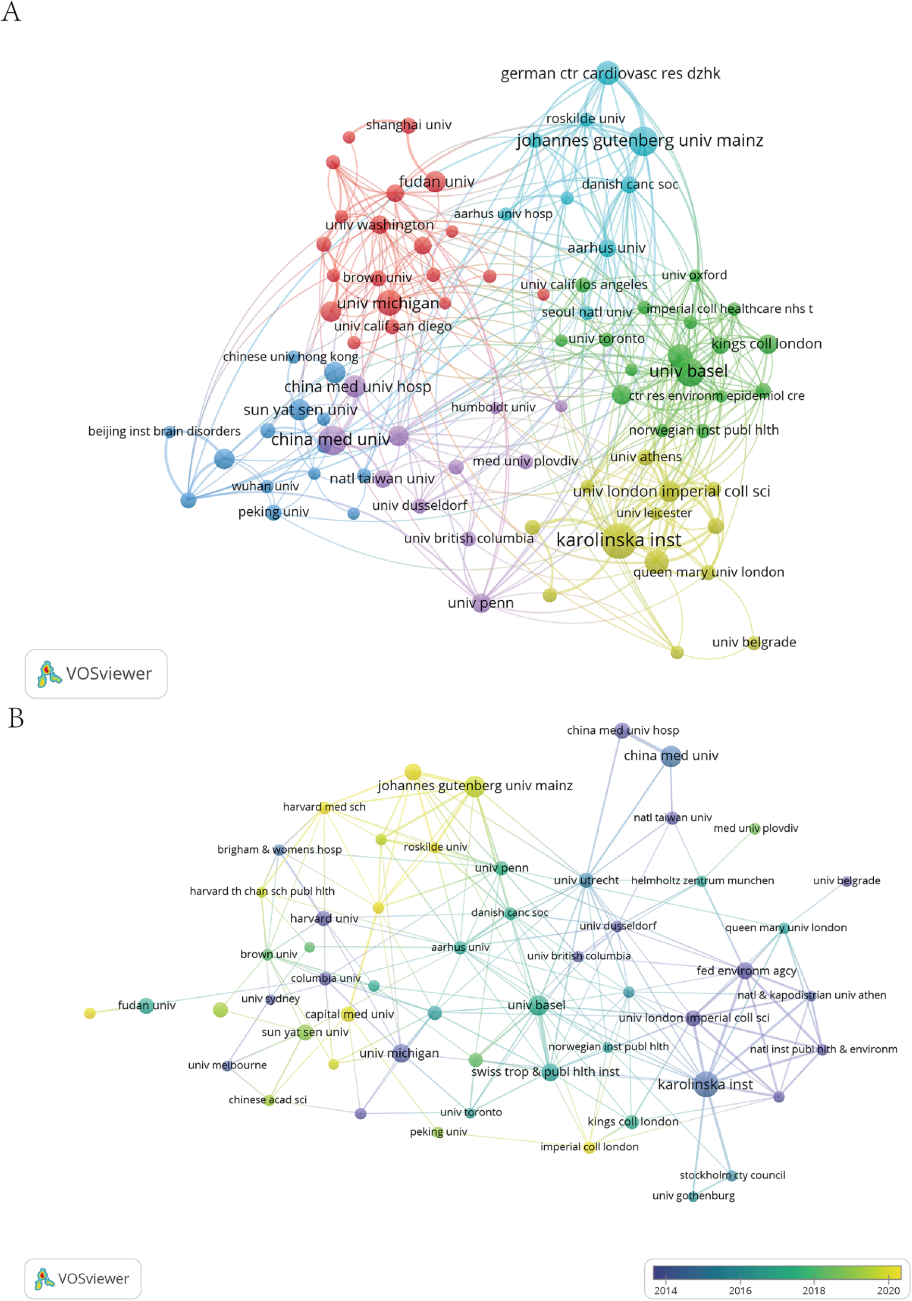
The network map of institutions. **(A)** Institutional cooperation network, illustrating collaborative relationships between institutions. **(B)** Evolution of the institutional network over time, showing the development and changes in institutional connections.

Research on hypertension and noise was carried out at approximately 1,540 institutions worldwide. The top 10 institutions, ranked by literature output and citation count, are listed in Table 3. Johannes Gutenberg Univ Mainz led with the highest number of publications (73, accounting for 7.29%), followed by Karolinska Inst with 60 publications (5.99%). The collaborative relationships among these institutions are depicted in Figure 5. Studies show that institutions with a higher number of publications and citations typically have more collaborative relationships with other institutions. This indicates that in the field of scientific research, institutions with substantial output and significant impact often attract more inter-institutional cooperation. Such collaboration not only enhances the quality and innovation of the research but also amplifies the institution's influence and recognition in the academic community.

**Table 3:** Top 10 productive institutions.

Institution	Publications	Citations
Johannes Gutenberg Univ Mainz	73	844
Karolinska Inst	60	1,269
China Med Univ	53	564
Huazhong Univ Sci & Technol	52	176
Capital Med Univ	35	19
Univ Basel	34	502
Univ Michigan	33	316
Kings Coll London	27	135
Univ Washington	26	209
Fudan Univ	25	96

### 3.5 Journals and co-cited academic journals

Analyzing the distribution of the sources of published articles is crucial for understanding the origins of research contributions. This analysis provides valuable insights for researchers, policymakers, and academic institutions in making informed decisions about collaboration, funding allocation, and strategic academic planning. A total of 1,001 articles related to this field were published in 437 academic journals. Table 4 lists the top 10 influential journals in this field. The journal *Noise & Health* (*n* = 35, 3.50%) has the highest number of publications, followed by *Environmental Health Perspectives* (*n* = 30, 3.00%), *International Journal of Environment* (*n* = 30, 3.00%), and *Environment International* (*n* = 27, 2.70%). Although *Noise & Health* had the most publications, it ranked third in citations. *Environmental Health Perspectives* had the most citations. A journal's influence is gauged by how often it is co-cited, indicating its importance within a specific research field. Among 8,777 co-cited journals, 13 were cited over 500 times. As shown in Table 4, the journal with the highest citation frequency was *Environ Health Perspective,* with 1,644 citations, followed by *Noise Health,* with 1,267 citations, and the *European Heart Journal,* with 852 citations.

**Table 4:** The top 10 journals with most publications.

Rank	Source	Documents	Citations
1	Noise & Health	35	1,188
2	Environmental Health Perspectives	30	3,349
3	International Journal of Environment	30	1,131
4	Environment International	27	1,520
6	Science of the Total Environment	22	954
7	Occupational and Environmental Medicine	17	979
8	Environmental Pollution	13	695
9	Environmental Science and Pollution	13	114
10	Environmental Health	12	525

**Figure 6:**
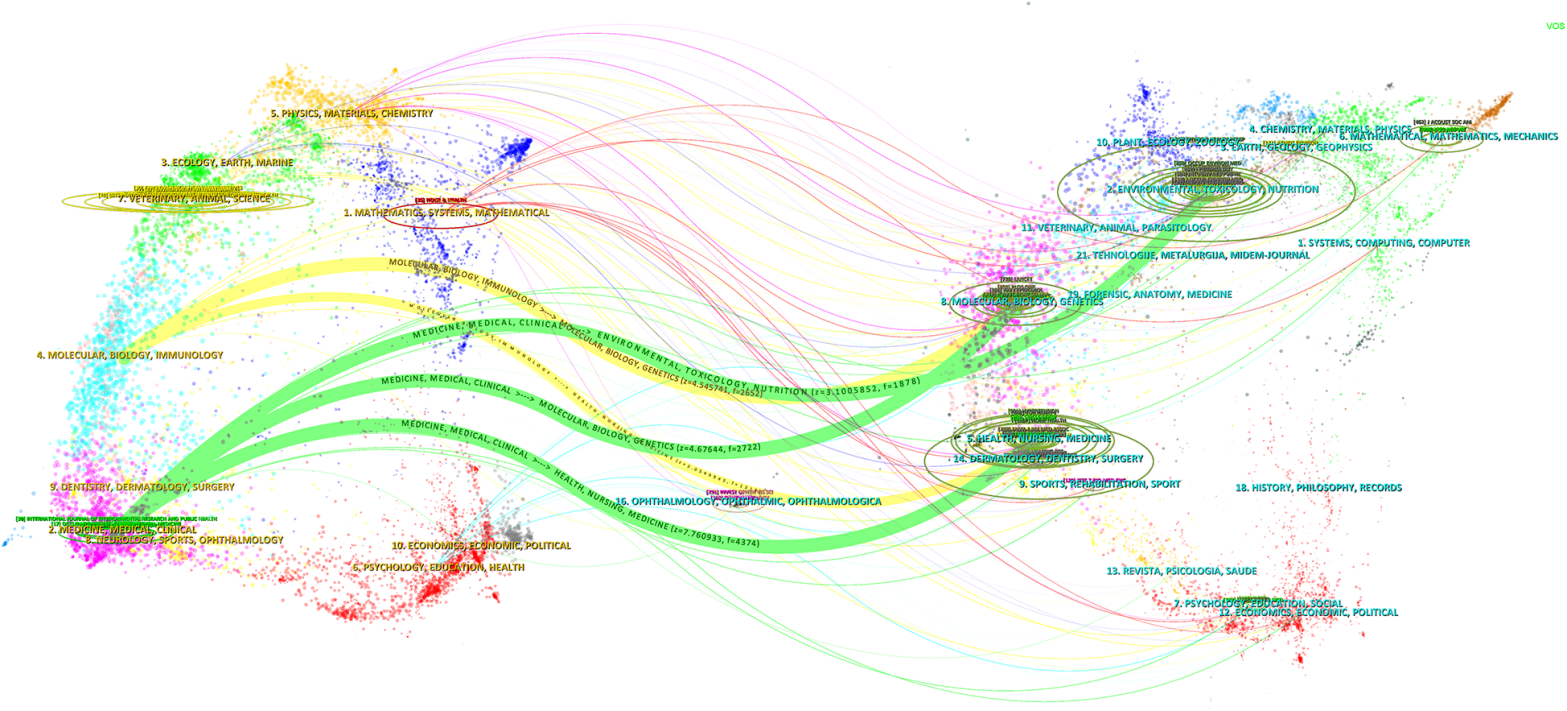
The dual-map overlay of journals. A dual-map overlay visualization showing the citation relationships between journals in two fields, with citation flows represented by colored arcs.

The dual-map overlay of journals provides a comprehensive visual representation of the relationships between different journals. In this overlay, journals that cite other works are displayed on the left side, while the journals that are being cited are shown on the right. The colored paths connecting these journals indicate citation relationships, effectively illustrating which journals reference others. As shown in Figure 6, there are five main citation paths, with three green paths and two yellow paths included. The green path indicates that publications published in Health/Nursing/Medicine, Environmental/Toxicology/Nutrition and Molecular/Biology/Genetics are cited for journals in Medicine/Medical/Clinical journals. The yellow path indicates that publications published in Health/Nursing/Medicine and Molecular/Biology/Genetics are cited for journals in Molecular/Biology/Immunology journals. This visualization is instrumental in understanding citation dynamics and the influence of various journals within a specific field. It offers a clear depiction of how research findings are disseminated through scholarly publications, thereby enhancing the analysis of citation patterns and the assessment of journal impact in the academic community.

### 3.6 Analysis of keywords and citations

**Figure 7:**
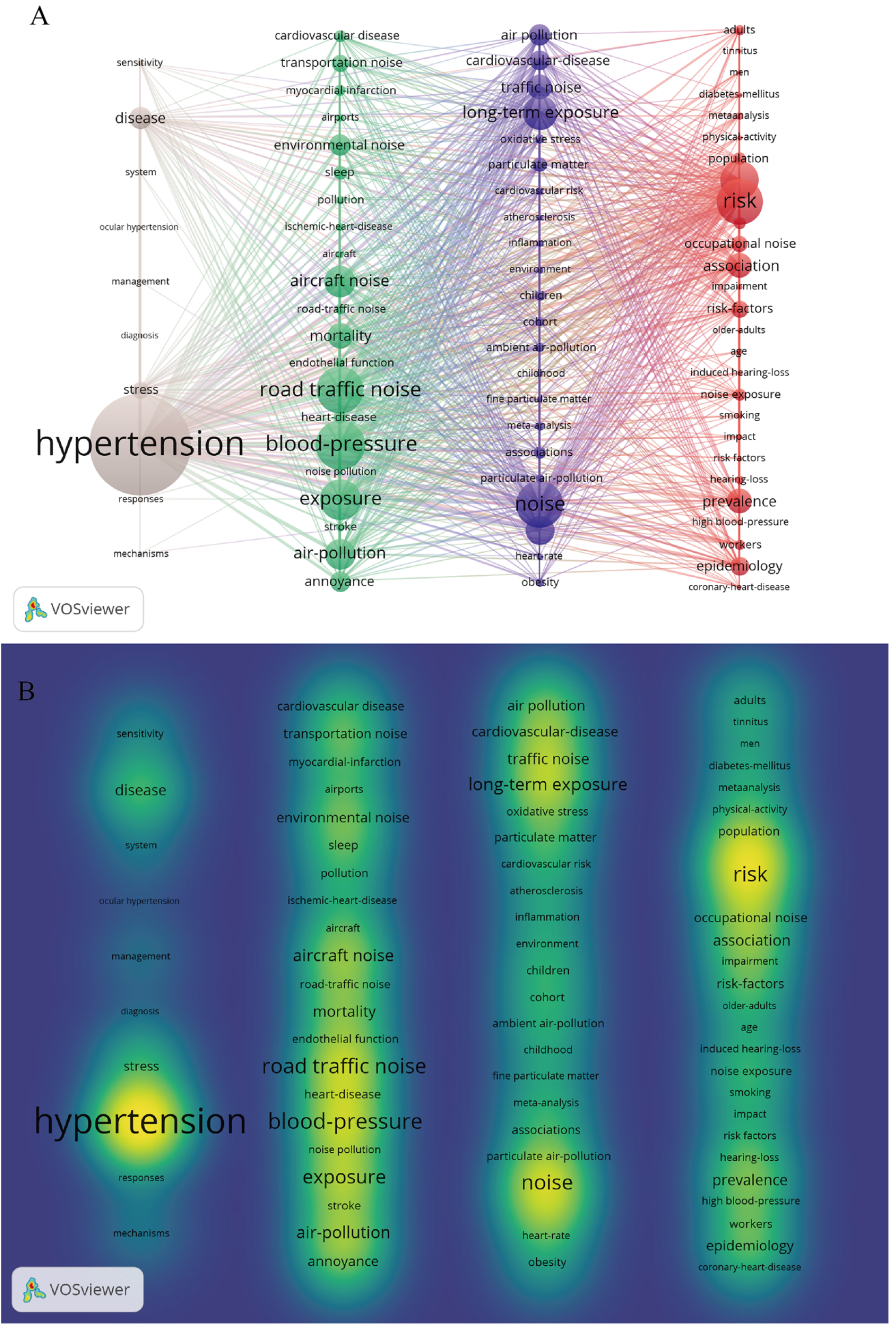
Keyword network analysis. **(A)** Network map of the top 100 keywords, organized into 4 clusters based on co-occurrence. **(B)** Density view highlighting the concentration of keyword relationships within the network.

**Figure 8:**
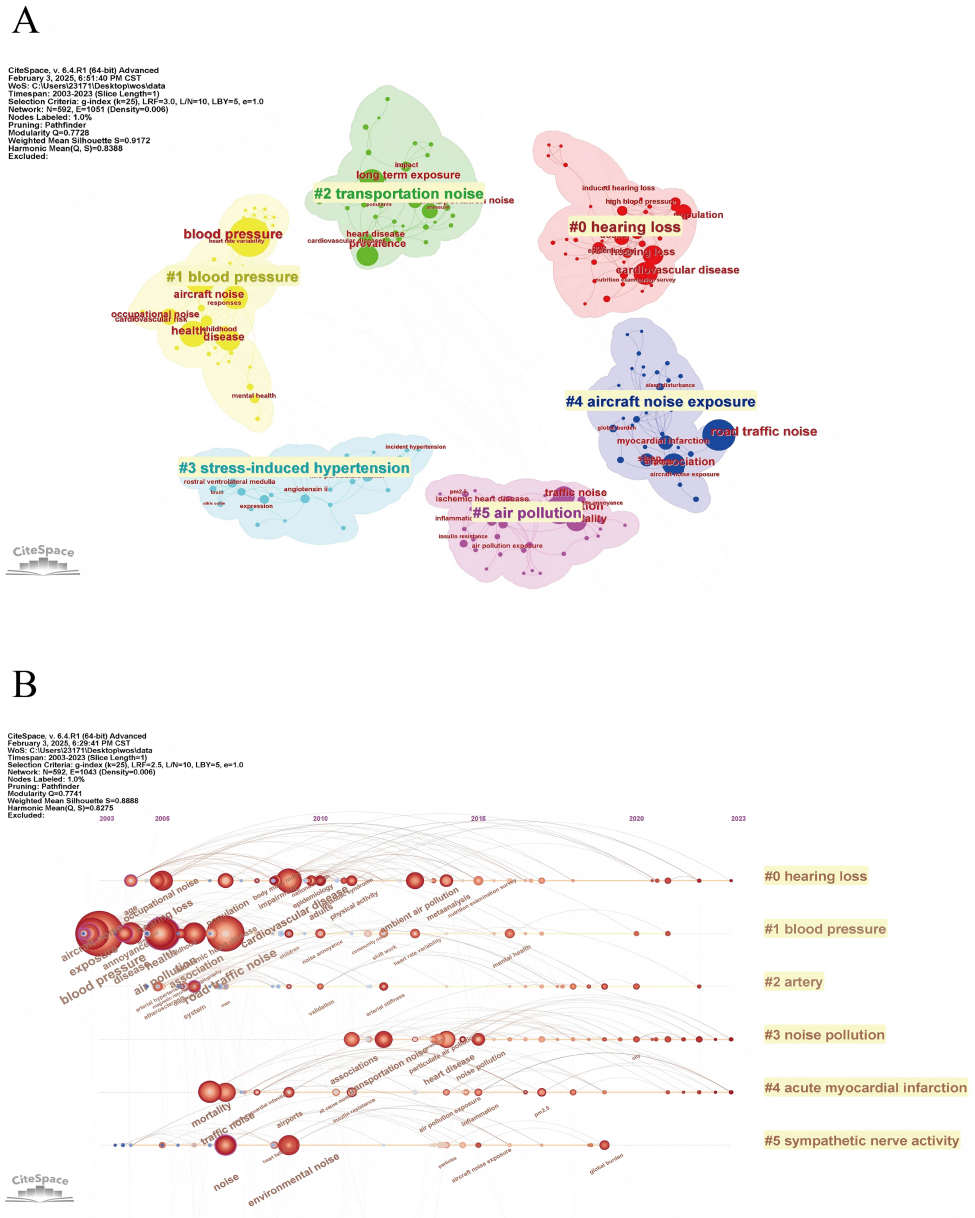
Keyword cluster analysis. **(A)** Cluster analysis of keywords conducted using CiteSpace. **(B)** Timeline view of the clusters, illustrating the evolution of keyword trends over time.

**Figure 9:**
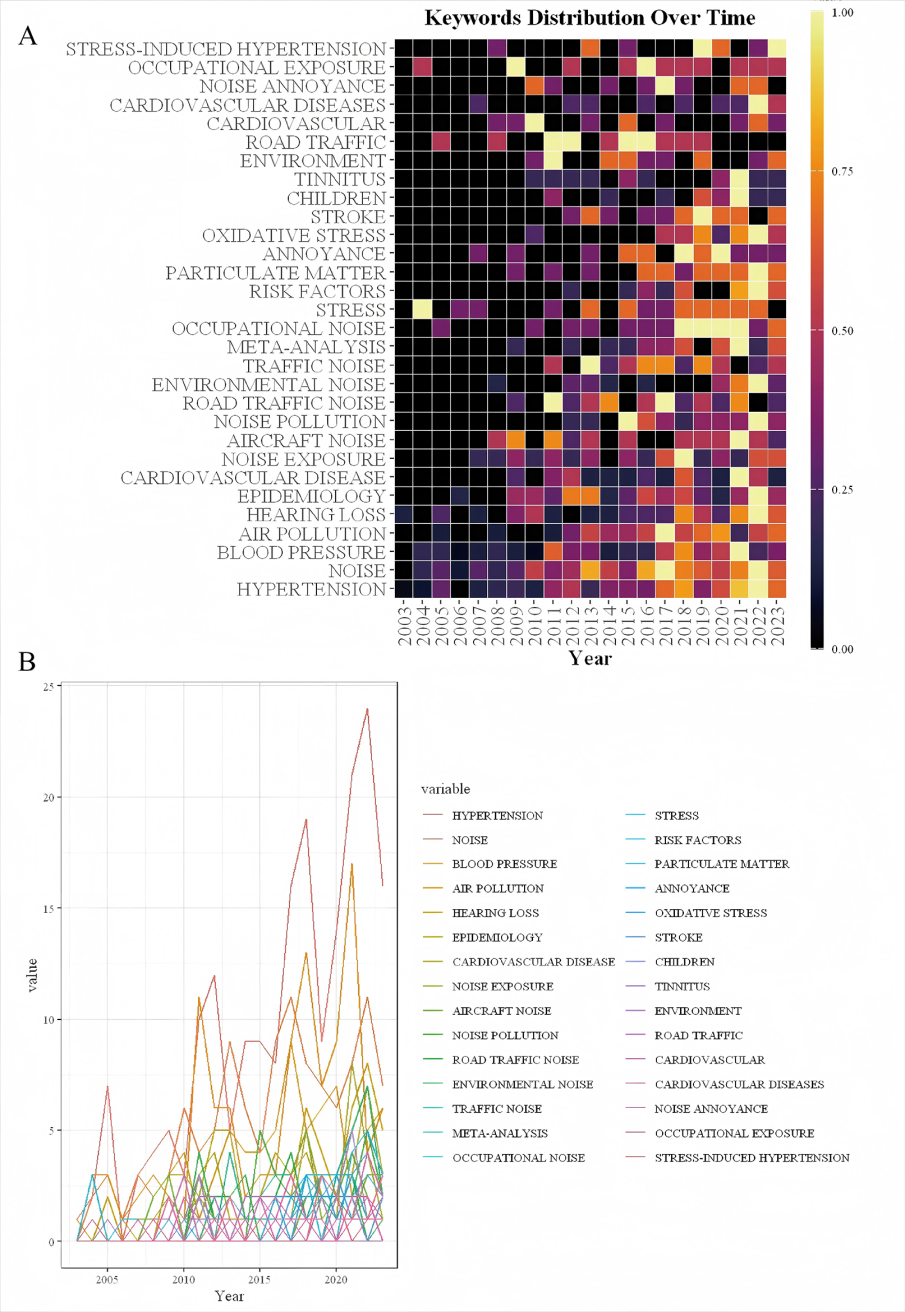
Keyword analysis using rtools. **(A)** Heatmap of keywords generated using Rtools, showing the frequency and intensity of keyword occurrences. **(B)** Line chart of keyword trends over time, created with Rtools, depicting the temporal evolution of keyword usage.

Keyword analysis plays a crucial role in bibliometric analysis, which involves examining the frequency and patterns of keywords within a collection of academic publications to discern emerging trends and core areas of research. It helps in identifying how topics evolve over time and revealing interdisciplinary connections and shifts in scholarly focus (13). From a total of 4,298 keywords across 1,001 papers, the top 100 most frequent keywords were extracted and clustered, with each occurring at least 14 times. Table 5 presents the top 10 keywords by frequency. Besides noise and hypertension, the five most frequent keywords were blood-pressure (216 occurrences), risk (196 occurrences), road traffic noise (176 occurrences), exposure (173 occurrences), and health (145 occurrences). As shown in Figures 7A,B, the VOSviewer-generated keyword co-occurrence map illustrates the relationships between keywords in research on hypertension and noise. The colors, positions, and line densities highlight the strength of associations and research hotspots. Different colors represent various thematic clusters or research hotspots. Brown cluster (e.g., “hypertension”) focused on hypertension-related research. Purple cluster (“long-term exposure,” “traffic noise”, et) related to long-term noise exposure and traffic noise. Green cluster (e.g., “road traffic noise,” “exposure”) associated with road traffic noise and exposure. Red cluster (e.g., “risk,” “epidemiology”) pertains to risk and epidemiology research. Figures 8A,B represent the co-citation network and the evolution of research topics over time. The chart is divided into 6 clusters, each representing a distinct research area. Cluster #0: Hearing Loss: Dominated by terms like “age,” “occupational noise,” and “hearing loss,” indicating a focus on how occupational noise exposure and aging contribute to hearing loss. Cluster #1: Blood Pressure: Keywords include “air pollution,” “association,” and “blood pressure,” reflecting studies on the impact of air pollution on blood pressure and related health outcomes. Cluster #2: Acute Myocardial Infarction: Terms such as “acute myocardial infarction,” “inflammation,” and “biomarkers” suggest research on the mechanisms and risk factors of heart attacks, with a focus on inflammation and biomarkers. Cluster #3: Noise Pollution: Includes terms like “noise pollution,” “air pollution,” and “heart disease,” highlighting the health impacts of environmental noise and air pollution. Cluster #4: Sympathetic Nerve Activity: Keywords such as “arterial pressure” and “sympathetic nerve activity” point to research on the physiological responses to noise and stress, particularly involving the sympathetic nervous system. Cluster #5: Magnetic Resonance Imaging: Terms like “arterial stiffness” and “magnetic resonance imaging” indicate studies using MRI to investigate cardiovascular health and arterial properties. The chart uses a color gradient to represent the temporal evolution of research topics, with colors ranging from blue (earlier years) to red (more recent years). Keywords like “air pollution” and “noise pollution” are prominent in recent years, reflecting increasing research interest in environmental factors affecting health. Newer terms such as “biomarkers” and “inflammation” indicate emerging areas of interest in understanding the biological mechanisms underlying the health effects of noise and pollution. Figure 9A visualizes the distribution of author keywords over time, with color intensity representing the frequency of keyword usage. Darker shades indicate higher usage frequency. Figure 9B shows the frequency of various keywords over time, providing a clear view of trends and fluctuations. Each line represents a different keyword.

**Table 5:** The top 10 keywords with the most occurrences.

Rank	Keyword	Occurrences
1	Hypertension	515
2	Blood-pressure	216
3	Risk	196
4	Noise	189
5	Road traffic noise	176
6	Exposure	173
7	Health	145
8	Aircraft noise	111
9	Long-term exposure	107
10	Air-pollution	103

**Figure 10:**
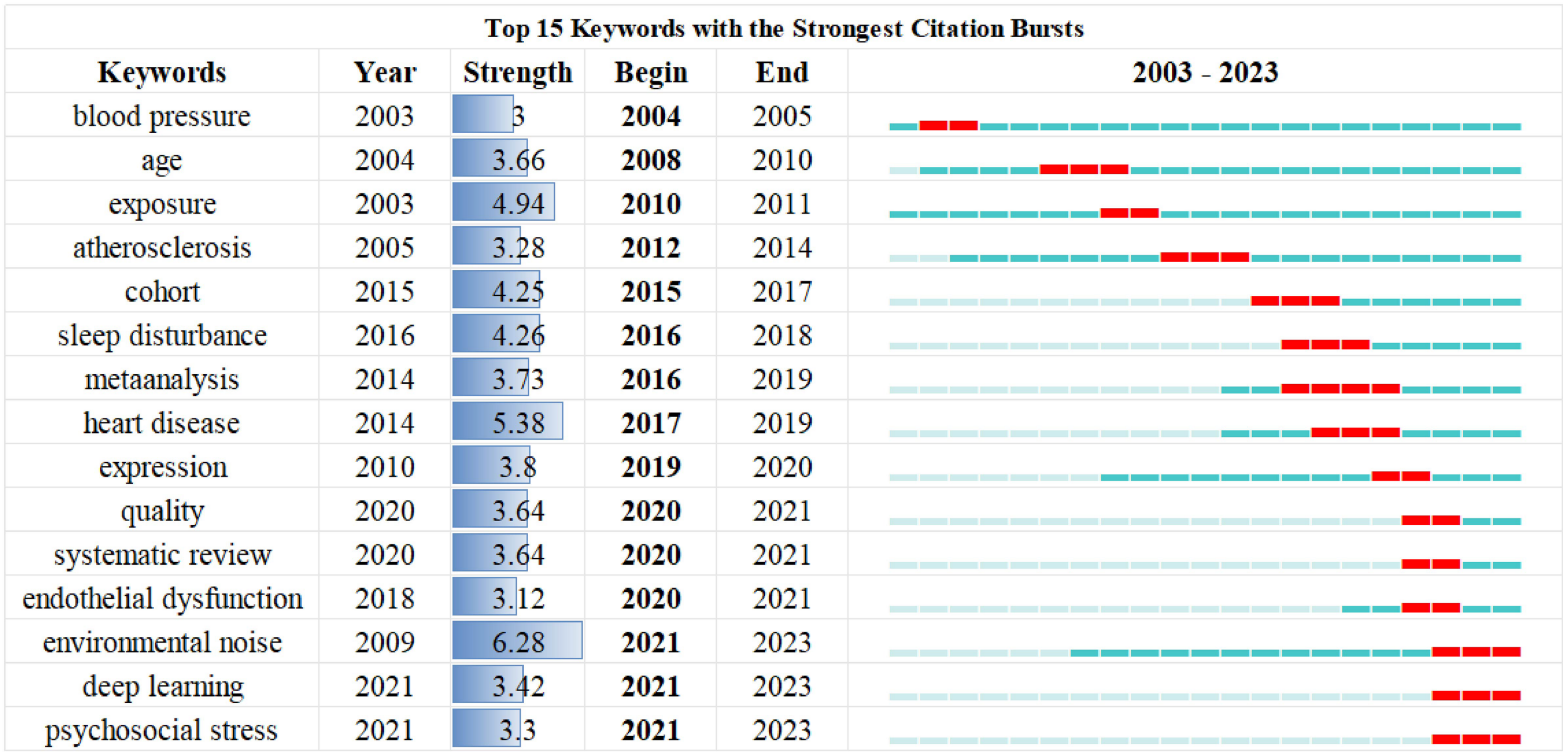
The top 15 keywords with the strongest citation burst. A visualization of the top 15 keywords exhibiting the most significant citation bursts, indicating rapid increases in citations over time.

Burst word analysis is a method used to identify and analyze keywords or topics that suddenly appear and quickly gain popularity in academic literature. This type of analysis helps researchers understand the emerging trends and shifts in research focus within a particular field (14). Figure 10 shows the 15 burst words identified in different periods in this study and reflects a shift from focusing solely on sources of noise in causing to mechanisms of noise-induced hypertension. These trends reflect the dynamic nature of health research and the importance of addressing emerging health challenges through focused scientific inquiry. Keywords “endothelial dysfunction”, “deep learning” and “stress” are emerging as hot topics in recent years.

**Figure 11:**
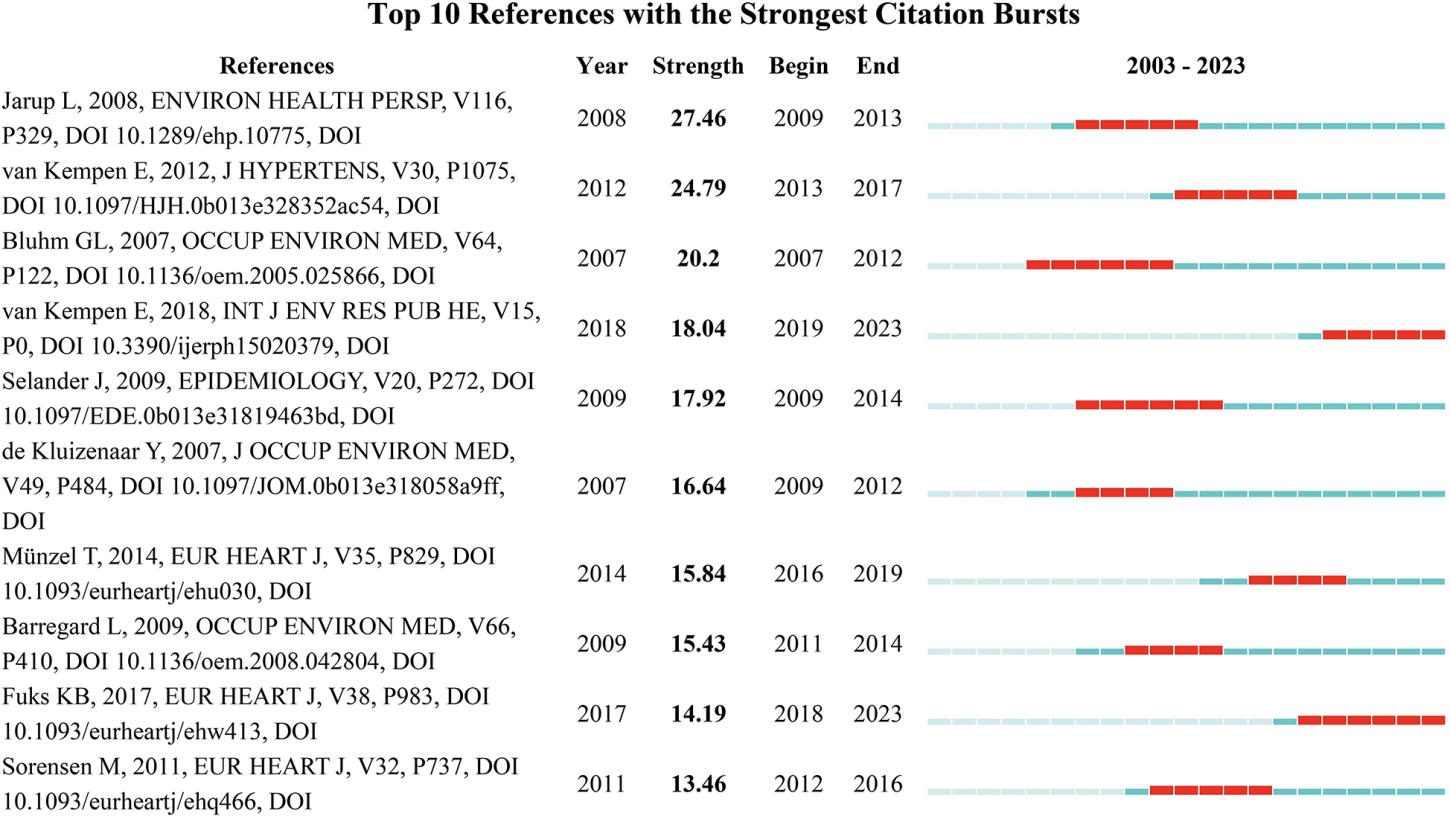
The top 10 cited papers in noise and hypertension.

Figure 11 presents the top 10 references with the strongest citation bursts, along with their corresponding burst periods. Citation burst strength reflects the intensity of a sudden increase in citation frequency for a reference during a specific period. The table provides details on the authors, publication year, journal name, volume, page numbers, and DOI for each reference. The reference with the highest citation burst strength is Jarup et al.'s 2008 article in Environmental Health Perspectives, which has a burst strength of 27.46 and a burst period from 2009 to 2013. Following closely is the 2012 article by van Kempen et al. in the Journal of Hypertension, with a burst strength of 24.79 and a burst period from 2013 to 2017. Notably, two articles published in 2007—one by Bhuhn et al. and the other by de Kluizenaar et al.—show significant burst strengths of 20.2 and 16.64, respectively, with burst periods from 2007 to 2012 and 2009 to 2012. These findings highlight the substantial impact these references had on research in their fields during those periods. Interestingly, van Kempen et al.'s 2018 article, despite having a relatively lower burst strength of 18.04, has a burst period extending through 2023, indicating its ongoing influence in recent research. Overall, the references with the strongest citation bursts are primarily concentrated in the fields of environmental health, occupational medicine, and cardiovascular diseases, reflecting the key research trends and hotspots during these periods.

## 4 Discussion

### 4.1 General information

Hypertension is one of the most common cardiovascular diseases worldwide. To present current research on global scientific output related to noise-induced hypertension, we utilized data from the Web of Science Core Collection (WoSCC) spanning from 2003 to 2023, employing bibliometric analysis as a novel technique to elucidate the knowledge structure in this field. Our findings indicate that this field has entered a golden era of research, as the volume of publications has witnessed a rapid increase since 2003, according to the trend line. The United States and China have much more publications than other nations/regions and are the most productive countries in this subject, according to analysis of country distribution. However, Germany stands out as the country with the highest research quality in this field, as it exhibits the highest average citation count for the papers produced in the region. According to the contribution and cooperation analysis among countries, China and the United States have engaged in extensive collaborations with numerous other countries, countries with more publications tend to have more cooperation with other countries, naming the significance of international cooperation, which can accelerate the development pace. Muenzel Thomas made the most papers and held major leadership roles in the field, and Noise & Health and Environmental Health Perspectives might be the main journals for this topic. Keyword and burst word analyses show that early research focused on noise source classification, while more recent studies have shifted towards investigating biological mechanisms such as endothelial dysfunction, circadian rhythm disruptions, and oxidative stress. This trend indicates a growing focus on the physiological pathways linking noise exposure to hypertension, signaling the shift from descriptive epidemiological studies to mechanistic research. By elucidating how noise triggers stress responses, disrupts sleep, and affects the autonomic nervous system, researchers can develop more effective intervention strategies. Fundamentally, bibliometric research provides a quantitative evaluation of the impact and visibility of scientific articles within the academic community. This involves analyzing citation counts, publication venues, and the overall impact of the research findings. Such an analysis helps assess the significance and reach of studies and identify research gaps, guiding future investigations.

Overall, the research is of significant value as it deepens our understanding of how noise exposure leads to hypertension, helps us know the research status and hot-spots within this area. This understanding is crucial for developing effective medical interventions and preventive measures. Additionally, the findings can help inform medical practitioners about the risks associated with noise pollution, thereby enhancing patient care and public health strategies. By understanding citation patterns and journal prominence, researchers can better evaluate the acceptance and significance of their work, ultimately contributing to the advancement of the field. The result of this research not only fosters improved medical interventions but also drives progress in the field. By enhancing our understanding of how noise exposure contributes to hypertension, the research leads to better health outcomes. Additionally, it advances knowledge about the impact of environmental factors on health, guiding future studies and informing public health policies aimed at reducing noise pollution and its harmful effects.

### 4.2 Mechanisms linking noise exposure to hypertension

Endothelial dysfunction is a major factor in noise-induced hypertension, as evidenced by its strong citation burst. The endothelium is a monolayer of cells lining the interior surface of blood vessels, playing a vital role in maintaining vascular homeostasis by regulating vascular tone, blood flow, and platelet activity (15, 16). Multiply studies have recognized noise as a risk factor for endothelial dysfunction (17–19). Moreover, endothelial dysfunction is closely related to hypertension indirectly (20, 21). Noise exposure triggers oxidative stress, leading to the overproduction of reactive oxygen species (ROS), which cause oxidative damage to endothelial cells and reduce nitric oxide (NO) bioavailability, impairing vasodilation (22, 23). This cascade disrupts vascular homeostasis and contributes to sustained increases in blood pressure. Additionally, noise-induced oxidative stress is thought to trigger inflammatory cascades, further damaging endothelial cells and exacerbating hypertension (24, 25). NO is a potent vasodilator produced by endothelial cells from L-arginine via endothelial nitric oxide synthase (eNOS). Noise-induced oxidative stress can also lead to the uncoupling of endothelial nitric oxide synthase (eNOS), an enzyme responsible for producing NO. This loss of NO bioavailability is a hallmark of endothelial dysfunction (18, 26). NO diffuses into the smooth muscle cells of the vessel wall, activating guanylate cyclase and increasing cyclic guanosine monophosphate (cGMP) levels, leading to vasodilation. This process is crucial for maintaining normal blood pressure and vascular tone (21, 27). Disruption of this process can be an adverse effector of hypertension. Noise exposure can also activate the HPA axis, leading to increased production of cortisol, a stress hormone. Elevated cortisol levels have been associated with impaired endothelial function due to their pro-inflammatory effects and the promotion of insulin resistance. Insulin resistance, in turn, contributes to endothelial dysfunction by reducing the bioavailability of NO and increasing oxidative stress (28). These factors ultimately contribute to vascular remodeling and impaired blood flow regulation, reinforcing the strong link between endothelial dysfunction and noise-induced hypertension. Our findings align with previous studies emphasizing endothelial dysfunction as a primary contributor to noise-induced cardiovascular risk. Hypertension and endothelial injury promote each other and are key factors leading to cardiovascular disease. Decreasing noise induced endothelial function may decrease the risk of the occurrence of hypertension.

Stress is a well-recognized factor contributing to the development and exacerbation of hypertension. As depicted in Figures 9A,B, stress-induced hypertension, starting from 2019, has gained heat in recent years. Among the various environmental factors that contribute to stress, noise is a significant yet often overlooked cause. Noise acts as a chronic environmental stressor, activating the sympathetic nervous system (SNS) and the hypothalamic-pituitary-adrenal (HPA) axis (29, 30). This leads to excessive catecholamine release, vasoconstriction, and elevated blood pressure. Noise-induced sleep disturbances further exacerbate stress-related hypertension by impairing physiological recovery (31–35). Bibliometric analyses indicate increasing research interest in this pathway, reinforcing its role in noise-related cardiovascular risk. Emerging evidence suggests that noise exposure can disrupt gut microbiota homeostasis such as small intestinal bacterial overgrowth (SIBO), glucose and lipid metabolism disorder and gut-brain axis disruption, leading to systemic inflammation and metabolic dysregulation (36–40). Noise-induced stress alters gut permeability and motility, promoting the proliferation of pro-inflammatory bacterial strains while reducing beneficial microbiota. This imbalance elevates systemic inflammatory cytokines, contributing to hypertension development (41–43). The growing focus on gut microbiota in noise research underscores its potential as a novel therapeutic target.

Noise exposure has been linked to sleep disturbances, which can interfere with circadian rhythms and have significant health consequences. Environmental noise, particularly at night, disrupts circadian rhythms, leading to dysregulation of blood pressure patterns (44–46). The Renin-Angiotensin-Aldosterone System (RAAS), which follows a circadian cycle, is affected by noise-induced sleep fragmentation. Alterations in circadian gene expression further contribute to hypertension, as disruptions in biological clock genes have been associated with increased cardiovascular risk (47–52). Recent bibliometric trends indicate rising research interest in this area, highlighting the need for further mechanistic studies.

### 4.3 Research gaps and future directions

Despite advances in understanding noise-induced hypertension, significant research gaps persist. Notably, our analysis reveals a lack of standardized methodologies for quantifying noise exposure, leading to inconsistencies across epidemiological and mechanistic studies. Future research should prioritize the development of internationally recognized noise assessment protocols to enhance cross-study comparability and reproducibility.

Moreover, while mechanistic studies have provided valuable insights, clinical translation remains limited. Multi-center cohort studies are needed to differentiate the effects of various noise types on hypertension subtypes. Additionally, targeted intervention strategies, such as antioxidants and sympatholytic agents, should be explored in clinical settings to mitigate noise-induced hypertension. The increasing role of artificial intelligence and machine learning in biomedical research suggests that predictive models could be developed to assess individual susceptibility to noise-related cardiovascular risks, enhancing precision medicine approaches in this domain.

### 4.4 Limitations

Our study has a few limitations. First, we exclusively used the WOSCC database and limited our selection to articles and reviews published in English, which may have caused some data omissions. Second, the analysis of publication numbers, citation metrics, and keyword trends could carry a degree of subjectivity, potentially leading to different interpretations by other researchers. Lastly, we cannot entirely ensure that every retrieved publication perfectly aligns with our search criteria. Despite these constraints, our study offers a broad perspective on the current landscape in this field.

## 5 Conclusions

The relationship between noise exposure and hypertension is a critical area of research that has gained substantial attention over the past two decades. Our bibliometric analysis highlights the significant growth in scientific output on this topic, particularly since 2016, with notable contributions from the United States, China, and Germany.

The core collaboration triangle formed by these countries highlights the essential role of international partnerships in advancing research into the mechanisms of noise-induced health effects. Early studies in this field primarily centered on the classification of noise sources, such as “road traffic noise” and “aircraft noise.” However, since 2016, there has been a marked shift toward molecular mechanism-based research, as evidenced by the surge in terms like “endothelial dysfunction” and “circadian rhythm.” This transition coincides with an overall increase in publication volume, signaling a deepening focus on underlying biological processes. “Endothelial dysfunction” has emerged as the most prominent keyword in recent years, with its co-occurrence closely linked to terms like “oxidative stress” and “NO bioavailability.” Furthermore, the rising interest in “deep learning” indicates a growing reliance on data-driven methods to unravel the molecular mechanisms at play. The consensus in the field is now clear: noise induces hypertension through the oxidative stress-endothelial injury pathway. Despite these advancements, current research remains predominantly focused on elucidating basic mechanisms, with limited clinical translation. A major limitation in the existing literature is the lack of standardized noise exposure metrics, leading to vague descriptions of noise dosage and hindering the reproducibility of mechanistic studies. Future studies should prioritize the development of standardized noise quantification methods. In addition, multi-center cohort studies are necessary to explore the associations between various types of noise exposure and hypertension subtypes. Targeted interventions, such as antioxidants or sympatholytic agents, should be developed, integrating clinical data to promote the translation of these findings into precision medicine.

## Data Availability

The original contributions presented in the study are included in the article/Supplementary Material, further inquiries can be directed to the corresponding authors.
